# Ping-Pong Free Advanced and Energy Efficient Sensor Relocation for IoT-Sensory Network

**DOI:** 10.3390/s20195654

**Published:** 2020-10-02

**Authors:** Moonseong Kim, Sooyeon Park, Woochan Lee

**Affiliations:** 1 Department of Liberal Arts, Seoul Theological University, Bucheon 14754, Korea; moonseong@stu.ac.kr; 2 Department of Electrical Engineering, Incheon National University, Incheon 22012, Korea; anisoo@inu.ac.kr

**Keywords:** mobile IoT, hopping sensor, sensory data networking, relocation protocol, energy-efficient protocol, ping-pong problem, simulation

## Abstract

With the growing interest in big data technology, mobile IoT devices play an essential role in data collection. Generally, IoT sensor nodes are randomly distributed to areas where data cannot be easily collected. Subsequently, when data collection is impossible (i.e., sensing holes occurrence situation) due to improper placement of sensors or energy exhaustion of sensors, the sensors should be relocated. The cluster header in the sensing hole sends requests to neighboring cluster headers for the sensors to be relocated. However, it can be possible that sensors in the specific cluster zones near the sensing hole are continuously requested to move. With this knowledge, there can be a ping-pong problem, where the cluster headers in the neighboring sensing holes repeatedly request the movement of the sensors in the counterpart sensing hole. In this paper, we first proposed the near-uniform selection and movement scheme of the sensors to be relocated. By this scheme, the energy consumption of the sensors can be equalized, and the sensing capability can be extended. Thus the network lifetime can be extended. Next, the proposed relocation protocol resolves a ping-pong problem using queues with request scheduling. Another crucial contribution of this paper is that performance was analyzed using the fully-customed OMNeT++ simulator to reflect actual environmental conditions, not under over-simplified artificial network conditions. The proposed relocation protocol demonstrates a uniform and energy-efficient movement with ping-pong free capability.

## 1. Introduction

Due to the development of IoT devices, it is possible to collect various types of data. Moreover, in areas where humans cannot access, small IoT devices are deployed using unmanned devices such as multirotor and drones to collect data [1,2]. However, since it is practically impossible to evenly place sensors in areas where data collection is required, it is challenging to collect appropriate data that can accurately reflect the characteristics of the area of interest. Even with proper placement, small IoT devices are likely to be defective due to the inherent problem of energy depletion [3,4]. The most crucial thing in big data and artificial intelligence technology is data analysis, and correct data collection must be continuously performed for accurate data analysis. However, even if a problem occurs in data collection due to defects in IoT devices, it is difficult to recognize abnormal data collection due to a large amount of data [5,6].

IoT sensor networking technology can be used to collect a variety of data from such as hard-to-reach military areas or radiation spill disaster areas with many obstacles. In order to analyze the characteristics of these areas, the sensors must be distributed evenly. If data collection frequently occurs in a specific area, sensors in this area are more likely to run out of energy faster than sensors in other areas, increasing the likelihood of device failure. In the worst case, this can cause the entire network to lose communication, making data collection impossible [7,8]. As such, an area where data collection is no longer possible is called **a sensing hole** [9].

Traditionally, energy consumption can be minimized by adjusting the state of the sensor, Active and Idle to prevent energy defects in the sensor node. Recently, methods based on energy consumption prediction have been proposed. For example, the prediction method of the sleep interval of sensors based upon the remaining battery level, previous usage history, and type of information required for an application is proposed [10]. Also, since energy consumption for message communication for controlling the terminal operation through node scheduling is significant, research is being conducted to optimize the idle time search and energy consumption model of the terminal through the fuzzy method [11].

In addition, various methods of establishing a path that can evenly consume energy when establishing a path for data transmission have been studied. However, these studies have not been able to overcome the fundamental energy defect problem of the sensor node [12,13]. The most reliable method is a sensor rearrangement method that restores the sensing hole by moving a sensor capable of collecting data to the sensing hole when a sensing hole occurs. The relocation method studied in the early days was a movement using wheels, but it had a limitation that movement was not free in rough terrain [14].

In order to overcome the disadvantages of the wheeled sensor, a hopping sensor that can jump and move to the desired direction was applied [15,16,17,18]. The initial hopping sensor relocation method assumed that all sensor devices knew information on all network areas, and then the sensor was moved [19,20,21,22,23,24,25]. However, this is a very inappropriate assumption in reality. To overcome this problem, our research team proposed a hopping sensor relocation protocol based on a distributed environment suitable for reality [25,26,27], but still, insufficiently researched topics such as mobile sensor requests were found.

In this paper, we explain and analyze the mentioned problem in detail, as shown in Table 1, and simulate the proposed relocation protocol using OMNeT++ for the first time in the world [28]. OMNeT++ is not a simple and easy-to-use network simulator but requires a lot of self-coding to simulate the real-world distributed environment.

The rest of our paper is organized as follows. Section 2 reviews the related study and mentions some problems for the previous work. Section 3 analyzes the problems and provides the advanced protocol to overcome the previous drawbacks. In Section 4, we have simulated to prove the better performance of the proposed scheme compared to the previous one. Finally, Section 5 concludes this paper.

## 2. Related Work

### 2.1. Descriptions of Hopping Sensors and Relocation Protocols

The feature of the hopping sensor is that it is easier to move in an area where obstacles such as rocks or sand exist by jumping rather than a wheel-based movement. In addition, it is possible to transmit data while jumping, thus widening the data transmission radius of the sensor. The authors of [16,17] explained that the sensor node could adjust the communication radius by jumping to the desired height. Also, it was shown that when the sensor node jumps 1 m from the ground, it increases about six times compared to the communication radius on the ground. In this way, the connectivity of nodes to the entire IoT sensor network can be made more robust. The authors of [18] actually implemented a hopping sensor using a separate launcher for jumping and measured the transmission radius.

It is not easy for randomly scattered sensors to place evenly over an area for data collection. Sensors may be concentrated in a specific cluster zone, and there may be cases that the number of sensors is insufficient to collect sufficient data in some areas. Sensors are depleted of energy due to continuous data collection, and this leads to states such as sensing holes that many sensors in the cluster zone cannot collect data due to energy failure. Therefore, a relocation protocol’s role is essential to supplement needed sensors from cluster zones with a sufficient number of sensors.

Using these hopping sensors, research on hopping sensors’ relocation to recover a sensing hole has been steadily progressing for decades. In [19], a method of relocating the sensors required for sensing hole recovery in the suppliable cluster zone on the shortest path was proposed. However, another sensing hole may easily occur because headers of a specific zone on the shortest path repeatedly participate in the role of a communication relay for relocation and additional relocation. Besides, there is a problem that some sensors rapidly lose mobile capability due to repeated movement. To solve this problem, in [21], multiple paths to replace the shortest path were used in the relocation strategy. As a result, it was possible to reduce the unbalanced movement distribution of the hopping sensors.

However, the critical problem in these studies is that all cluster headers set the relocation path in real-time with all current information of the entire network. In fact, no matter how small the entire network area, information exchange and path setting between all cluster headers is practically impossible, and numerous control messages are generated. In other words, the relocation policy in a distributed environment is the most realistic method rather than source-based relocations. Recently, our research team has solved this source-based problem [25,26,27]. First, all sensor nodes do not need to know the information of neighboring sensor nodes and the entire network. It is a relocation protocol based on distributed networking similar to reality by requesting sensor nodes to nearby cluster headers to recover sensing holes. The next section introduces basic terms and assumptions and introduces the most representative distributed environment-based relocation protocol.

### 2.2. Basic Assumptions of Hopping Sensor Networking in a Distributed Environment

**Figure 1:**
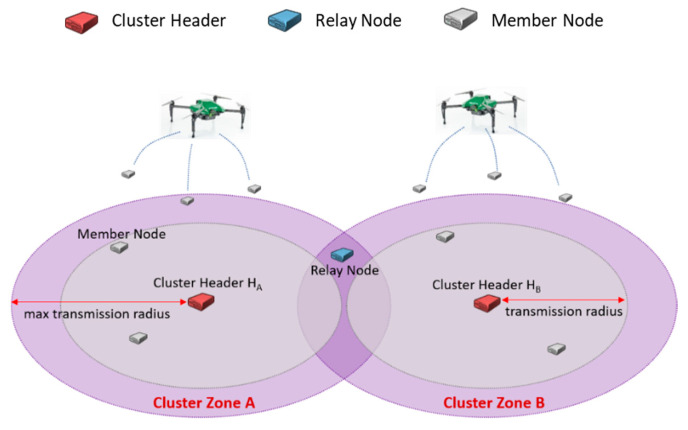
The terms for IoT hopping sensor networks.

IoT hopping sensors are randomly distributed to a specific area to collect interesting data. For instance, sensors can be placed using UAVs and drones in areas where humans cannot access, as shown in Figure 1. The area could be divided into several appropriate cluster zones with well-known clustering algorithms. A proper sensor in each cluster zone’s center is elected as a cluster header, and the remaining sensors in the same zone as each header become member nodes. The header communicates periodically to manage information about its member nodes. This paper assumes that clustering and header selection methods are already available using various algorithms [29], and it omits further discussion. Furthermore, the hopping sensor can jump to communicate with neighboring sensors, and the sensors can know their location using GPS [30]. The hopping sensor’s characteristics have been known in previous papers, so detailed explanations are also omitted.

Figure 1 briefly explains the terms used in this paper. Every hopping sensor node can be **a cluster header** or **a sensor member node**. When an elected cluster header is jumping highest, the maximum transmission area is defined as a cluster zone; it may be impossible to communicate between cluster headers directly. There is a possibility that some sensor member nodes in the area intersecting the neighboring cluster zones can communicate with more than one cluster header. In other words, the intersected area may be near the maximum transmission radius of each cluster header. We call this sensor member node as **a relay node**, and the role of the relay node has to serve to help the communication between cluster headers.

### 2.3. The Previous Relocation Protocol and the Proposed Protocol’s Contributions

**Figure 2:**
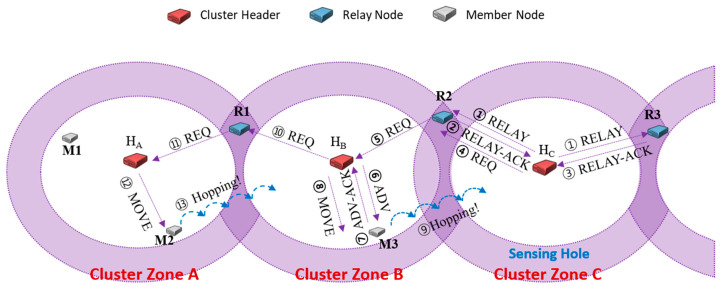
An example of hopping sensors relocation [25].

When a sensing hole occurs due to an insufficient number of sensor member nodes in a cluster area, the sensing hole’s header has to request the relocation of needed sensor member nodes from the neighbor cluster zones. In this case, a relay node plays an intermediate role in delivering some messages between some neighbor cluster zones. In Figure 2, when the header H_C_ of cluster zone C detects the sensing hole, H_C_ first broadcasts a RELAY message to its relay nodes R2 and R3 (①). Each of R2 and R3 replies RELAY-ACK message to H_C_ (②, ③), respectively. If H_C_ first receives the RELAY-ACK message from R2, it transmits a REQ message containing the number of needed sensor member nodes (here, the number is 1) to R2 (④). The selected relay node R2 immediately delivers the received REQ message to the header H_B_ of cluster zone B (⑤). H_B_ selects its sensor member nodes (here, M3 is selected) as many as the number of requested nodes recorded in the REQ message. H_B_ broadcasts an ADV message to its zone to check the movable sensor nodes (⑥). Upon receiving the ADV message, the member nodes send an ADV-ACK message to their headers containing their information (current coordinates, energy residuals, etc.) (⑦). H_B_ selected the appropriate members (in our simulation, a policy is used to select member nodes which are close to the neighbor header), and it transmits MOVE messages for moving to the sensing hole to them (⑧). The sensor member node receiving the MOVE message migrates to cluster zone C, and the sensing hole could be recovered (⑨). However, H_B_ predicts that cluster zone B will become a sensing hole and immediately selects relay node R1 and requests the necessary sensors (10–13), as performed by the header H_C_ (⑩–⑬).

In the example of Figure 2, the number of members to recover sensing holes in all cluster zones is one. Assuming that only cluster zone C needs two sensors, the header H_B_ could be requested to relocate two sensors. In the distributed environment, relay nodes cannot know how many current members are in cluster zone B. A relay node merely forwards the received REQ message to its header. As a concern, cluster zone B currently has only one member, not two or more; thus only one sensor, which is able to move currently, is selected and migrated. At the same time, the header H_B_ also predicts that its zone could be a sensing hole and immediately requests as many as the needed sensors from the neighboring cluster zone A.

Although the mentioned relocation protocol [25] can recover sensing holes occurred, there is a possibility that the following problems happen.

- In Figure 2, the MOVE message’s destination address is the GPS coordinate of the header H_B_. Therefore, a phenomenon occurs in which the relocated sensors are concentrated around the header H_B_. Sensors of a cluster zone must be placed evenly to collect representative data of the cluster zone. However, in the previous relocation protocol, the relocated sensors are inevitably moved around the header due to the cluster header’s GPS information.
- Whenever a sensing hole of cluster zone B occurs in Figure 2, the header H_B_ has to broadcast a RELAY message. If a RELAY-ACK message from the relay node R1 among response messages of relay nodes always arrives first due to the shortest distance-based manner, the cluster header H_B_ continuously requests needed sensors to the cluster zone A to recover its sensing hole. However, if a sensing hole also occurs in cluster zone A, every request of the header H_B_ continues to fail; it may not be easy to recover the sensing hole.

Table 1 summarizes these problems and the solutions for the problems in this paper. In the next section, detailed explanations of case analysis and solutions for each problem are introduced.

## 3. The Proposed Relocation Protocol for Ping-Pong Free

In this section, we look at the mentioned problems through a case study in detail, and we propose a relocation protocol to overcome the ones.

### 3.1. The Relocation Scheme to Evenly Distribute Sensors

**Figure 3:**
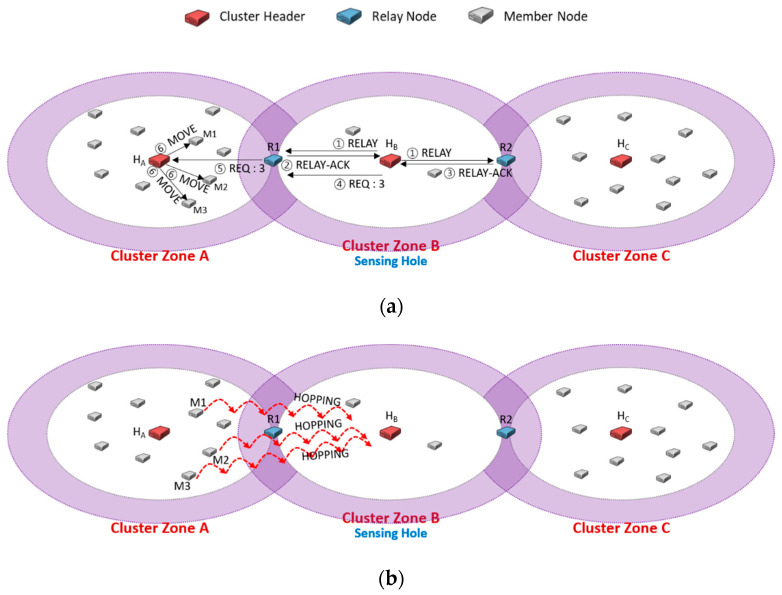
An example of the problem that the moved sensor nodes are relocated near the header of the sensing hole, H_B_.

Figure 3 shows an example of the problem that sensor member nodes relocated to recover the sensing hole are around the cluster header. Let us consider that at least five sensor member nodes have to be retained to recover a sensing hole and adequately collect data. As shown in Figure 3a, we assume that the cluster zones A and B have ten and two nodes. As soon as the header H_B_ detects its sensing hole, it sends RELAY messages to relay nodes R1 and R2 (①). The relay nodes R1 and R2 reply to H_B_ with RELAY-ACK message, respectively (②, ③). If H_B_ first received RELAY-ACK from R1, it sends REQ messages to R1 and then ignores RELAY-ACK from R2. Here, it adds the information of the needed number of sensor nodes to recover the sensing hole to the REQ message. The relay node R1 forwards the received REQ message to the header H_A_ in cluster zone A (⑤). The header H_A_ chooses appropriate three sensor members and sends MOVE messages to them to migrate to the sensing hole (⑥).

In the previous scheme [25], the MOVE message contained GPS coordinate information of the sensing hole header’s destination. In Figure 3b, the member nodes M1, M2, and M3 that have received MOVE messages may migrate to the header H_B_’s adjacent area. As shown in Figure 3b, this migration would overcome the sensing hole with five members of cluster zone B. However, it would not be able to collect data reflected representative characteristics of cluster zone B as a whole. It would be desirable to modify GPS information in MOVE messages so that the members who move as much as possible could be distributed evenly. Thus, a coordinate (*x, y*) of the destination H_B_ has to be modified as Equation (1):

(1) $$\left(x,y\right)=\left(x_{H}\pm rand\left(0,r\right)cos\theta ,\;y_{H}\pm rand\left(0,r\right)sin\theta \right)$$

where (*x_H_, y_H_*) is a coordinate of a destination cluster header, rand(a, b) is a function generating an arbitrary number between two real numbers a and b, r is a maximum transmission radius, and θ is a direction of movement from a member node to a destination cluster header.

### 3.2. The Relocation Scheme to Uniformly Choose Relay Nodes for Ping-Pong Free

**Figure 4:**
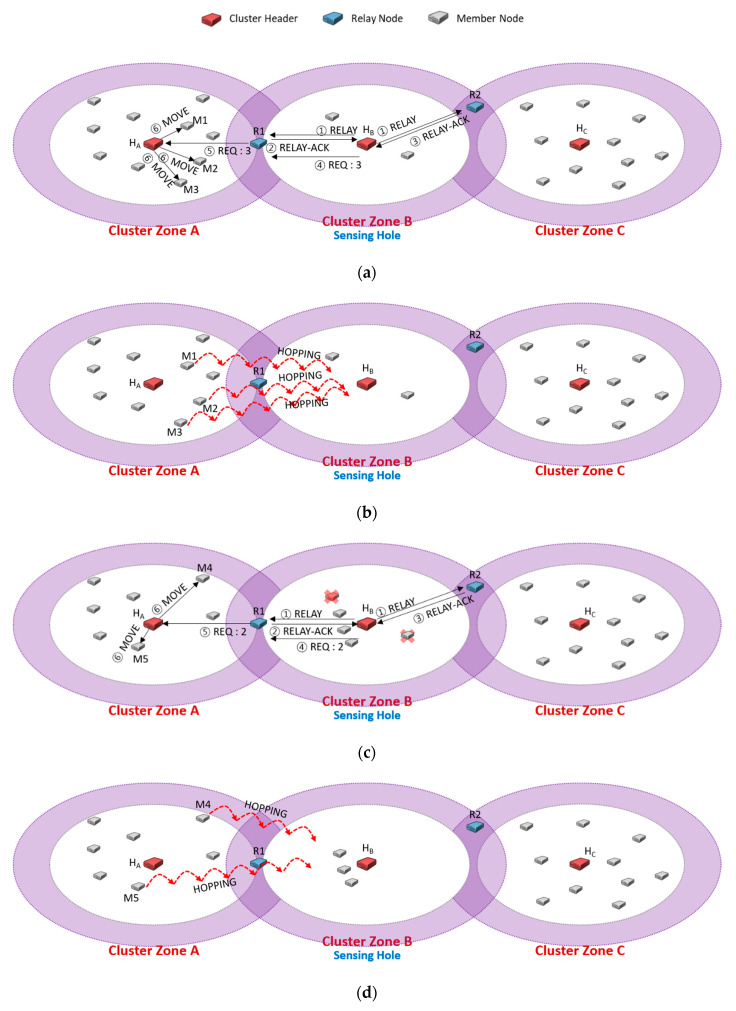
An example of continuously sending a message to request the needed members only to a specific relay node, R1.

Figure 4 shows the case of continuously sending a message to request the needed members only to a specific relay node. We assume that there are three cluster zones, and cluster zone B is a sensing hole. A cluster zone is determined as a sensing hole if the number of member nodes is less than 5. Figure 4a shows that the header H_B_ of the sensing hole broadcasts RELAY message to relay nodes R1 and R2 (①). Upon receiving the RELAY message, the relay nodes R1 and R2 reply RELAY-ACK messages to H_B_, respectively (②, ③). Suppose that H_B_ first received the RELAY-ACK transmitted from R1. H_B_ adds the information of the number of members (i.e., 3) to the REQ message and transmits it (④), and then R1 delivers the message to the header H_A_ (⑤). Finally, H_A_ sends MOVE messages to three appropriately selected nodes among all members (⑥).

Suppose that two members are exhausted of energy in cluster zone B, so the sensing hole occurs again, as shown in Figure 4c. To recover the sensing hole, H_B_ broadcasts RELAY message to relay nodes R1 and R2 (①). As explained in Figure 4a (②~⑤), R1 forwards the REQ message to H_A_ to relocate some needed members. In the relocation protocol of [25], as each relay node replies RELAY-ACK message as soon as it receives the RELAY message from its header, it could be regarded as the shortest distance-based protocol. The reason is that the purpose of the previous relocation protocol is to pursue a short recovery time. However, this has to request some needed members to a specific relay node, and nodes relocation continuously appears in a duplicated neighbor cluster zone. Therefore, although the sensing hole could be recovered quickly, there is a disadvantage in which the neighbor zone can be quickly turned into a sensing hole. As shown in Figure 4d, cluster zone A would become a sensing hole if one more request happens.

**Figure 5:**
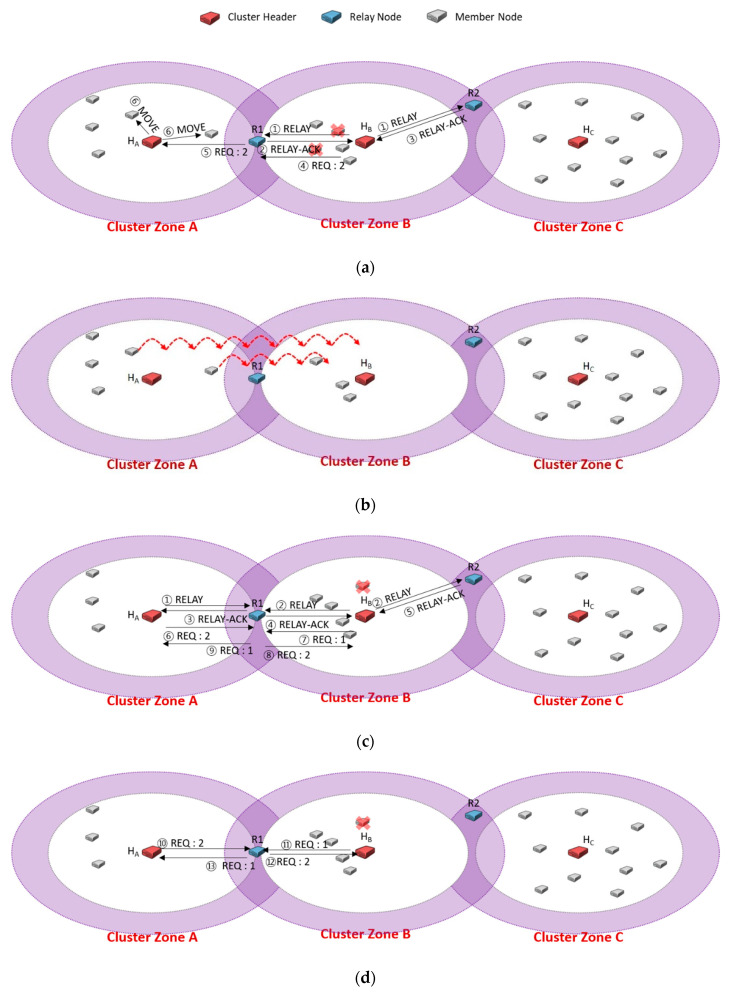
A case study of the ping-pong problem.

Figure 5 describes the ping-pong problem such that two neighboring cluster zones are sensing holes, and each cluster header continuously requests the needed members to the same relay node. As shown in Figure 5a following Figure 4d, cluster zone B is a sensing hole, so the cluster header H_B_ transmits an REQ message, including the number of needed sensors (i.e., 2) to the relay node R1 (④). The neighbor cluster header H_A_ relocates appropriate members to the sensing holes (⑥), and the sensing hole could be recovered in Figure 5b.

Just in time, cluster zone B becomes a sensing hole again due to a node failure in Figure 5c. Another sensing hole also occurs due to some member relocation of Figure 5b. Each cluster header (i.e., H_A_, H_B_) chooses the relay node R1 to request the required member (①~④). Although each cluster zone is a sensing hole, relay node R1 delivers the REQ message received from H_A_ to H_B_, but H_B_ ignores the received REQ message because its cluster zone is a sensing hole (⑥, ⑧). Besides, the relay node R1 forwards the REQ message received from H_B_ to H_A_, but the H_A_ ignores the received REQ message since its zone is also a sensing hole (⑦, ⑨). As a result, the recovery of each sensing hole fails. After a certain period (i.e., HELLO message interval time), as shown in Figure 5d, the header of each sensing hole selects the relay node as R1 again for recovering each sensing hole (HA: ⑩, ⑫, HB: ⑪, ⑬), it could fail to recover them like Figure 5c.

**Figure 6:**
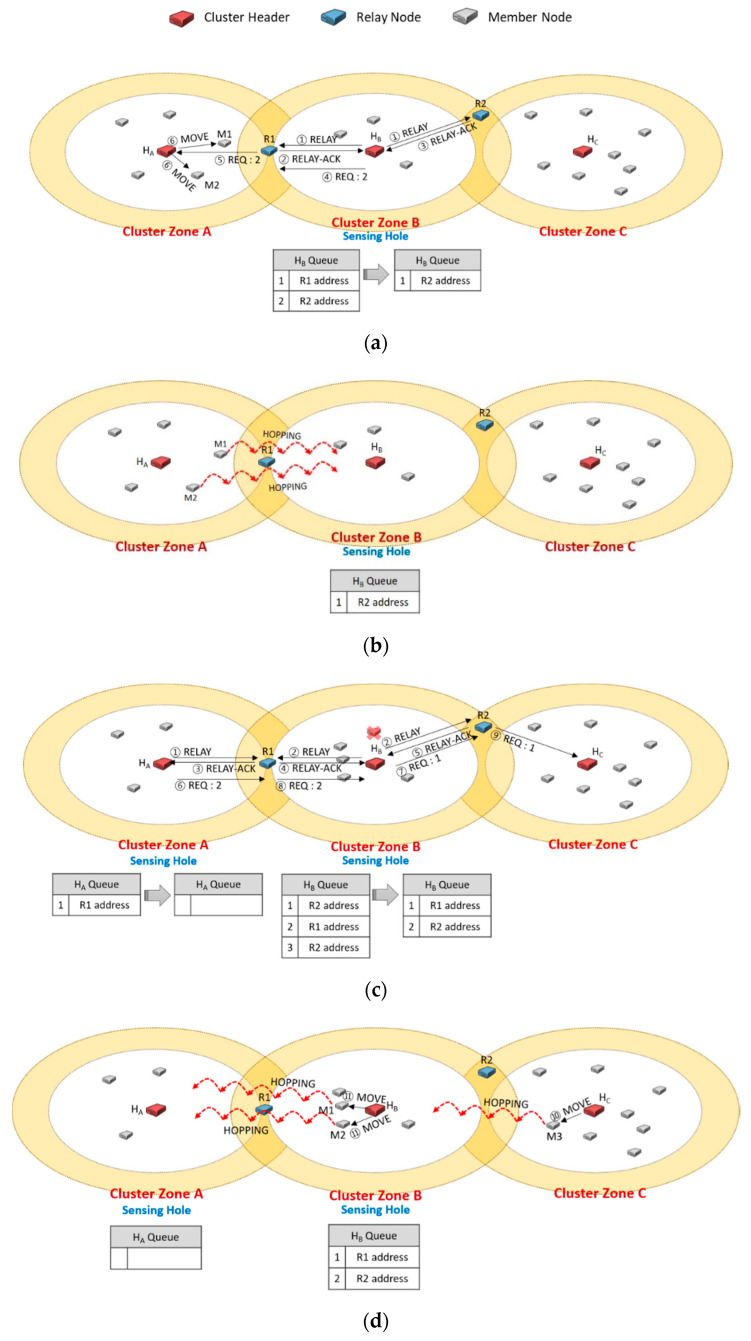
An example of using each header’s queue to manage the priority of selecting a relay node for ping-pong free.

Undoubtedly, the method to solve the ping-pong problem as described in Figure 5d is to evenly choose a relay node, not the shortest distance-based selection. As shown in Figure 6, we proposed a queue-based scheme to select a relay node equally in this paper. Each header adds the relay nodes as candidates to its queue using RELAY-ACK messages in order. Each candidate node will be brought out in order of priority when a cluster header chooses a relay node.

In Figure 6a, the cluster header H_B_ of cluster zone B detects a sensing hole and broadcasts a RELAY message (①). Each relay node responds with a RELAY-ACK message as soon as receiving RELAY. Here, if H_B_ received the messages from R1 and R2 sequentially (②, ③), it adds the addresses of R1 and R2 to its queue in order. H_B_ sends a REQ message contained the required number of members (i.e., 2) to the priority R1 and deletes the address of R1 from the queue. H_A_ received the REQ message (⑤), and it orders M1 and M2 to move to the neighbor cluster zone B through MOVE messages (⑥).

In Figure 6b, cluster zone A becomes a sensing hole as the members move. In Figure 6c, cluster zone B is also a sensing hole due to a member node’s failure. The header H_A_ broadcasts a RELAY message (①), and another header H_B_ also broadcasts another RELAY message in its zone (②). In cluster zone A, H_A_ puts the address of R1 in its queue as receiving a RELAY-ACK message from R1 (③). In cluster zone B, H_B_ also adds the addresses of R1 and R2 to its queue when it receives RELAY-ACK messages from R1 and R2 (④, ⑤), respectively. In order to select an appropriate relay node, each cluster header dequeues a candidate relay node with a higher priority in its queue. So, H_A_ selects R1 and transmits a REQ message (⑥), and R1 delivers the message to H_B_ (⑧). H_B_ dequeues the R2 address and sends a REQ message to R2 (⑦). R2 forwards the message to H_C_ (⑨). Therefore, as shown in Figure 6d, the ping-pong problem could be prevented in advance.

In Figure 6a, suppose that the RELAY-ACK message (③) was not received from the relay node R2, which is far away due to network communication failure, and the RELAY-ACK message (⑤) was received only in Figure 6c. In this case, since the address of R1 enters the queue twice in a row, ping-pong may occur, as shown in Figure 5. In fact, the intention of the FIFO policy is to evenly select the relay nodes of the candidate group and evenly request the neighboring headers to move the required hopping sensor. However, in an environment where such an abnormal situation may occur, after sequentially putting the addresses of relay nodes into the queue, managing the frequency of the addresses entered in the queue is separately performed, and relay nodes for the minimum frequency can be selected. In this way, it is possible to solve the ping pong problem that may occur through communication failures such as the example mentioned now. Alternatively, the FIFO can be replaced in a variety of ways, such as using a random function or a hash function when selecting from the queue, or shuffling the addresses of relay nodes in the queue. In this way, various policies other than FIFO can be used for queue-based relay node candidate management, but this paper assumes that the most well-known FIFO method is applied.

**Figure 7:**
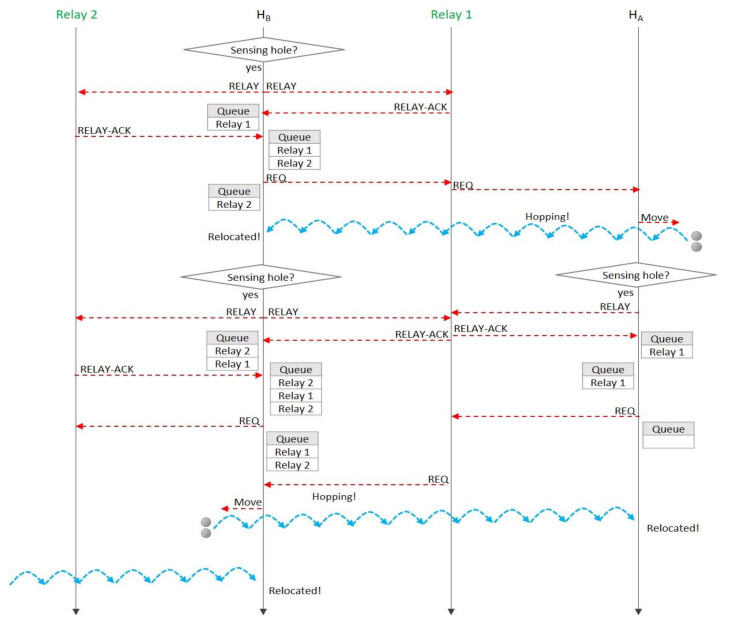
A message sequence diagram for the case study of Figure 6.

Figure 7 is a sequence diagram of the proposed algorithm for Figure 6. We can look at the queue’s changing over time, and it could explain the proposed relocation protocol in detail.

## 4. Simulation Results and Analysis

In the past, the relocation algorithm of mobile sensors was only a theoretical study. The simulation also had an assumption that every sensor node was aware of all changed topologies. It could not implement the consideration in reality. One of the contributions of this study is that a distributed networking-based relocation protocol is proposed to advance the mentioned limitations (Table 1) of the previous work, and the simulation is performed using OMNeT++ to increase the possibility of implementation in the real world [28,31,32]. Table 2 describes the simulation environment.

**Figure 8:**
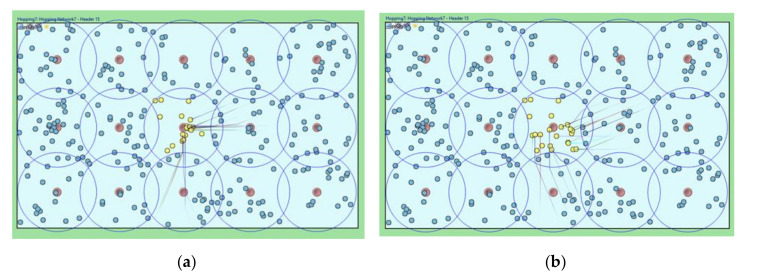
Simulation snapshots of the movements of sensor member nodes. (**a**) Previous scheme; (**b**) Proposed scheme.

300 sensors are scattered randomly, as shown in Figure 8; If necessary, the sensors can be distributed in a particular way, but they are generally uniformly distributed in our simulation. Among them, 15 cluster headers are arranged (red color), and the remaining 285 things are sensor member nodes (i.e., 19 member nodes per cluster zone). We consider the scenario in which every sensor member node continuously collects requested data in the central cluster zone and rapidly consumes energy. We set a simulation parameter that each sensor in the middle cluster can generate a data collection event with exponential distribution (average of 5 min). A cluster header can detect an occurrence of a sensing hole (less than ten sensor member nodes) after broadcasting a HELLO message for every specific period (i.e., HELLO message interval time). We also color a sensor member node as yellow when it becomes faulty due to energy depletion after continuous data collection. We indicate the movement of a sensor member node by a solid line.

Figure 8a shows that the previous relocation algorithm moves required sensor member nodes around the sensing hole header since the destination of the moving sensor is set to the GPS coordinate of the header. However, Figure 8b shows the relocation of the required sensor member nodes through the proposed algorithm in which the destination address of the MOVE message is reset to a random value near the header. As can be seen, the moved sensor member nodes are evenly arranged in the sensing hole area, and appropriate data could be collected.

**Figure 9:**
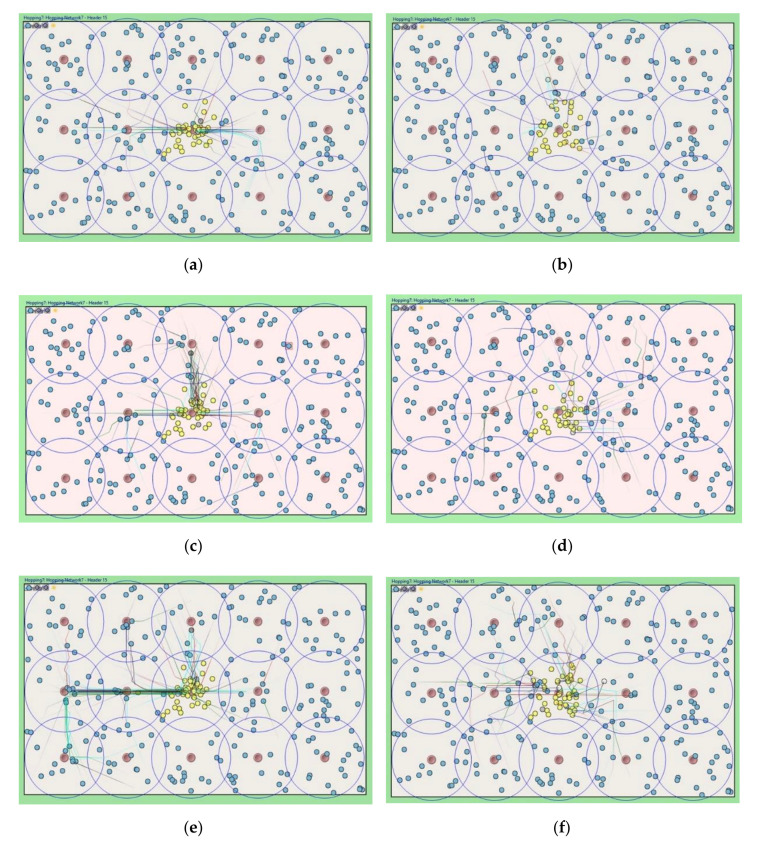
Simulation snapshots for each relocation protocols in terms of HELLO message interval time. (**a**) Previous scheme w. 60 min; (**b**) Proposed scheme w. 60 min; (**c**) Previous scheme w. 30 min; (**d**) Proposed scheme w. 30 min; (**e**) Previous scheme w. 15 min; (**f**) Proposed scheme w. 15 min.

The cluster header periodically broadcasts a HELLO message to detect that its zone becomes a sensing hole. We set the periodic time to 60, 30, and 15 min, and we considered the simulation time was three days. Figure 9a,c,e show the simulation snapshots of the problem of sending a REQ request message to specific relay nodes in the previous algorithm, as mentioned in Section 3.2. As shown in Figure 9b,d,f, most relay nodes are uniformly involved in the relocation process. The proposed algorithm can evenly move most sensor member nodes and lead to uniform total network energy consumption.

**Figure 10:**
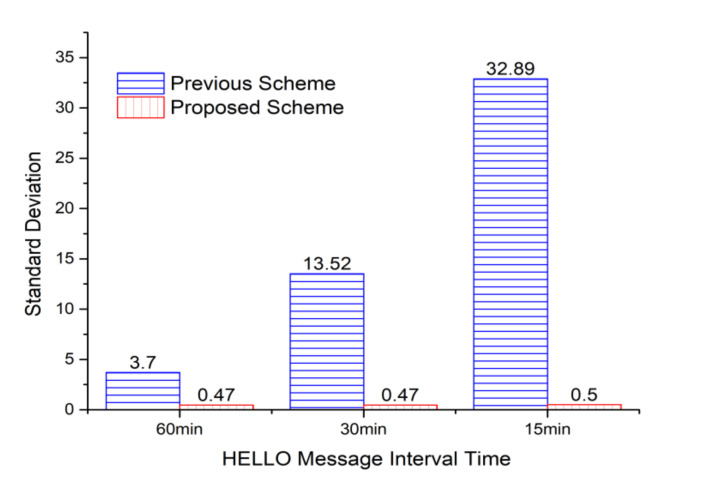
Standard deviations of the numbers of relay nodes selected by the middle cluster header in Figure 9.

Figure 10 shows the standard deviation values for the frequency of selecting relay nodes to request the needed sensors from the cluster header, which is the middle of the area in Figure 9. Since the header selects a relay node that responds fastest in the previous algorithm, there is a very high possibility that specific relay nodes could be repeatedly selected. It is also improbable that the relay nodes located in the remote place are selected in response to late. In the proposed algorithm, the priority of the relay nodes to be selected is managed as a queue, so the standard deviation value for the frequency of selecting relay nodes is relatively low compared to the previous one. In other words, as can be seen in Figure 9, it is confirmed that the majority of relay nodes are selected in a similar proportion.

**Figure 11:**
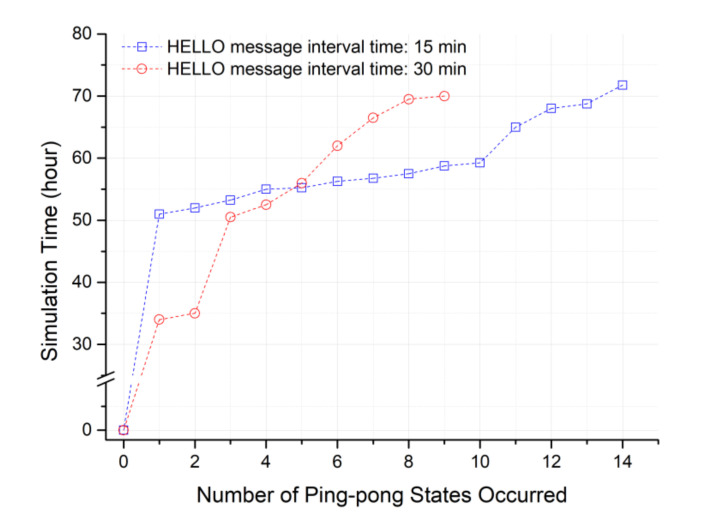
Occurrence time of ping-pong states in terms of the number of ping-pong states for the previous scheme (currently the proposed scheme has NO ping-pong in this simulation.).

In the proposed scheme, there was almost no occurrence of a ping-pong phenomenon. However, as shown in Figure 11, the ping-pong phenomenon steadily occurred in the previous scheme due to using specific relay nodes as close as possible. When the HELLO message interval of the cluster header capable of detecting the sensing hole is set to 15 min, there is a cluster zone in which continuous data collection is actively performed. In this case, a continuous sensing hole may occur, which means that a ping-pong state is likely to occur. In Figure 11, it can be seen that many ping-pong problems occurred between 50 and 60 h of simulation.

However, in Figure 11, the ping pong of the proposed method could not be examined. As shown in Figure 10, this is because relay nodes are selected almost evenly in the proposed method. In other words, the standard deviation does not exceed 0.5 times for the number of times the relay nodes are selected. Next, an additional simulation was performed by configuring an extreme environment in which a ping-pong state may occur, and additional energy consumption to solve the ping-pong problem was analyzed. Table 3 describes the environment settings.

**Figure 12:**
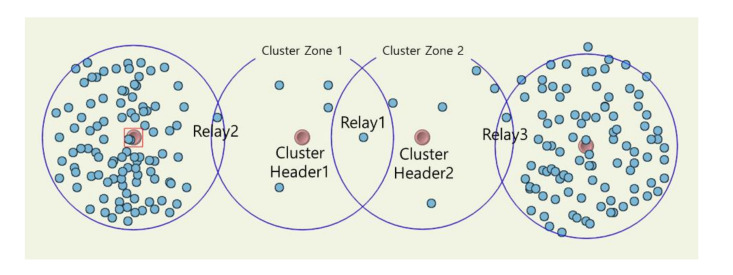
Simulation snapshot for generating ping-pong states.

**Figure 13:**
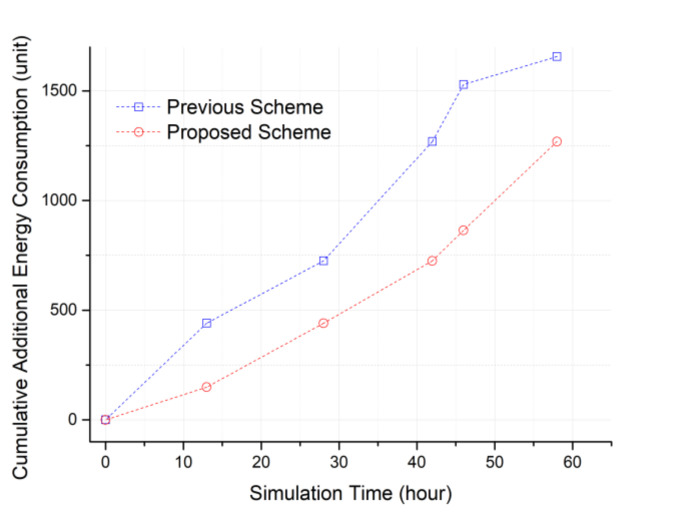
Cumulative additional energy consumptions to resolve the ping-pong states occurred.

Figure 12 is a topology where a lot of ping-pong can occur. Relay node 1 is in the middle between the two cluster headers, and relay nodes 2 and 3 are a little farther apart. Cluster zones 1 and 2 are in a state where the maximum number of sensing holes can occur. In this topology, relay node 1 will be selected in the previous method, but in the proposed method, it can be predicted that relay nodes are sequentially selected. Once ping-pong occurs, many additional messages are generated again. At the moment which the sensing hole cannot be recovered, RELAY, RELAY-ACKs as much as the number of relay nodes, 2 REQs, ADV, ADVs as much as the number of members in the cluster zone, and MOVE as the number of selected members are additionally generated as seen in Figure 2. In Figure 13, even in such an extreme environment, the proposed method achieved a maximum energy efficiency of about 43.5% compared to the previous method after performing the simulation for about 40 h.

When the ping-pong phenomenon occurs, the sensing hole’s recovery time may be delayed, and as a result, it becomes difficult to collect data continuously. Furthermore, the continuous generation of REQ messages occurs, and it may incur serious energy consumption throughout the entire networking area. Therefore, we are confident that our advanced relocation protocol can effectively extend the network lifetime compared to the previous one.

## 5. Conclusions

Our research team has previously proposed practical sensor relocation protocols for mobile sensors used in real-world environments. However, it was found that sensors in the specific cluster zones near the sensing hole were continuously requested to relocate. Thus, there can be an unbalanced sensor selection and ping-pong problem, and as a result, fast energy exhaustion makes the overall sensor network lifetime shorter.

The proposed algorithm, designed by a fully-customed OMNeT++ simulator, first focuses on a uniform selection of the sensors to be relocated. We could confirm that the proposed relocation protocol showed a uniform and energy-efficient sensor movement as possible compared to previously proposed algorithms. Thus, a more uniform distribution of sensors can reflect the characteristic of the zone better. It also expects to reduce energy consumption by saving send-messages to request the necessary sensors. Then, the ping-pong problem was solved by the proposed protocol based on the queue-dequeue process using the queue considering the priority of the request.

## Figures and Tables

**Table 1:** Limitations of the previous relocation protocol [25] and contributions of this paper.

Limitations	Proposed Schemes’ Contributions
· The destination of moving sensors is the GPS coordinates of the cluster header of the sensing hole. · After relocation, the relocated sensors are locally distributed around the sensing hole header; thus, only data around the header can be collected.	· The destinations of moving sensors are randomly separated from the cluster header of the sensing hole. · After moving, it is evenly distributed in the cluster zone; thus, it is possible to collect data from the overall zone.
· The cluster header selects the relay node that responded first among response messages arriving from the relay nodes; thus, it is highly possible that the only several relay nodes are selected. · There is a high possibility that a message ping-pong with a neighboring zone occurs.	· The header manages relay nodes by using queues. · With various policies such as FIFO, relay nodes are selected evenly, so that mobile nodes move evenly throughout the network. · There is a low probability of message ping-pong with neighboring zones.

**Table 2:** Simulation environments.

Network Area	250 m × 150 m
number of all hopping sensor member nodes	285
number of cluster headers	15
minimum number of members for each cluster to properly gather data (i.e., a sensing hole occurs if the number of current members lower than this value)	10
maximum communication radius for each sensor node	20 m
maximum communication radius when highly jumping	29 m
maximum distance that a sensor node moves forward with one jump	2 m

**Table 3:** Simulation environments for generating ping-pong states.

Network Area	250 m × 60 m
number of all hopping sensor member nodes	285
number of cluster headers	4
minimum number of members for each cluster to properly gather data	5
maximum communication radius for each sensor node	20 m
maximum communication radius when highly jumping	29 m
maximum distance that a sensor node moves forward with one jump	2 m
Simulation time	3 days
